# 52 million years old *Eucalyptus* flower sheds more than pollen grains

**DOI:** 10.1002/ajb2.1569

**Published:** 2020-12-03

**Authors:** Maria C. Zamaloa, Maria A. Gandolfo, Kevin C. Nixon

**Affiliations:** ^1^ Museo Paleontológico Egidio Feruglio Avda. Fontana 140 Trelew, Chubut 9100 Argentina; ^2^ LH Bailey Hortorium Plant Biology Section School of Integrative Plant Science Cornell University Ithaca NY 14853 USA

**Keywords:** Argentina, early Eocene, fossil, Myrtaceae, *Myrtaceidites*, Patagonia

## Abstract

**PREMISE:**

Fossils provide fundamental evidence of the evolutionary processes that crafted today’s biodiversity and consequently for understanding life on Earth. We report the finding of *Myrtaceidites eucalyptoides* pollen grains preserved within the anthers of a 52‐million‐year‐old *Eucalyptus* flower collected at Laguna del Hunco locality of Argentinean Patagonia and discuss its implications in understanding the evolutionary history of the iconic Australian genus *Eucalyptus*.

**METHODS:**

Pollen grains were extracted from the flower’s anthers and were then observed under light microscopy and scanning electron microscopy. The phylogenetic position of the fossil was investigated by adding pollen data to a previously published total‐evidence matrix and analyzing it using parsimony.

**RESULTS:**

We erect the species *Eucalyptus xoshemium* for the fossil flower. Pollen extracted from *E. xoshemium* belongs to the species *Myrtaceidites eucalyptoides*, which, until now, was only known as dispersed pollen. The numerous pollen grains recovered from the single flower allowed estimation of *M. eucalyptoides*’ variability. Results of the phylogenetic analysis reinforce the position of this fossil within crown group *Eucalyptus*.

**CONCLUSIONS:**

The discovery of these pollen grains within a Patagonian *Eucalyptus* fossil flower confirms the hypothesis that *Myrtaceidites eucalyptoides* represents fossil pollen in the *Eucalyptus* lineage, extends the geographic and stratigraphic fossil pollen record, and supports an earlier age for crown‐group eucalypts.

Fossils provide fundamental evidence of the evolutionary processes that crafted today’s biodiversity and for understanding life on Earth. Furthermore, with the advent of molecular dating of phylogenies, fossils have become even more critical because they are undoubtedly the only concrete evidence of life in deep time (Marshall, 1990; Nixon, 1996; Crepet et al., 2004; Benton and Donoghue, 2006; Clarke et al., 2011; Morlon et al., 2011, Silvestro et al., 2014; Magallón et al., 2015; Betts et al., 2018; Hopkins et al., 2018). They provide crucial information for assessing homology and evolutionary changes because they offer character evidence that affects phylogenetic conclusions and thus our awareness of modern relationships, corroborate past distributions that can aid in comprehending biogeographic history and distribution patterns, and are essential for estimating minimum ages of the clades to which they belong and to all the nodes directly ancestral to the clade in which they are placed (Gandolfo et al., 2008). Unfortunately, most fossils are incomplete and often lack preservation of critical features. This is exacerbated for fossil plants because the majority of the organs are found isolated and only occasionally in organic connection, and because of the biased nature of fossil preservation, i.e., soft and delicate organs and/or parts such as flowers and petals are less often preserved than are hard parts such as wood and leaves (Stewart and Rothwell, 1993; Gandolfo et al., 2008, Taylor et al., 2009; Xing et al., 2015). Nonetheless, compressed fossil flowers and fruits are sometimes exquisitely preserved (e.g., Crepet and Daghlian, 1981; Basinger and Dilcher, 1984; Crepet and Taylor, 1985; Sun et al., 2002; Friis et al., 2011) providing suites of characters that allow them to be studied in great detail and placed phylogenetically.

During the last 15 years, persistent fieldwork in Argentinean Patagonia has yielded several superbly preserved fossil flowers and fruits (Zamaloa et al., 2006; Gandolfo et al., 2009, 2011; Futey et al., 2012; Hermsen et al., 2012; Hermsen and Gandolfo, 2016; Gandolfo and Hermsen, 2017; Jud et al., 2017, 2018a, b; Wilf et al., 2018, 2019), which in many cases represent the oldest records globally for the taxa and the clades they belong. Among these, Gandolfo et al. (2011) and Hermsen et al. (2012) reported a diverse and beautifully preserved suite of fossils belonging to the iconic Australian genus *Eucalyptus*. The fossils were collected at the early Eocene (~52 mya) sediments that outcrop at Laguna del Hunco locality, Chubut Province, Patagonia, Argentina (Fig. 1). The fossil *Eucalyptus* remains include leaves and infructescences, as well as isolated capsules, flower buds, and two flowers preserved at different developmental stages. Although none of the fossils were preserved in organic connection, each one has synapomorphies that independently support their assignment to *Eucalyptus* sensu stricto. Gandolfo et al. (2011) confirmed the taxonomic position of the fossils as they explored their relationships with extant members of the tribe Eucalypteae (the “eucalypts”) and *Eucalyptus* species by including them in a phylogenetic study. The taxa considered in their phylogenetic study included Patagonian fossils as a single terminal plus all the members of the monophyletic tribe Eucalypteae, which is composed of seven genera: the monotypic *Allosyncarpia* S.T. Blake, *Stockwellia* D.J. Carr, and *Arillastrum* Pancher ex. Baill., *Eucalyptosis* (with 2 species), *Angophora* Cav. (13 species), and the largest *Corymbia* K.D. Hill & L.A.S. Johnson (113 species) and *Eucalyptus* (with more than 600 species) (Ladiges et al., 2003; Parra‐O et al., 2009).

**Figure 1:**
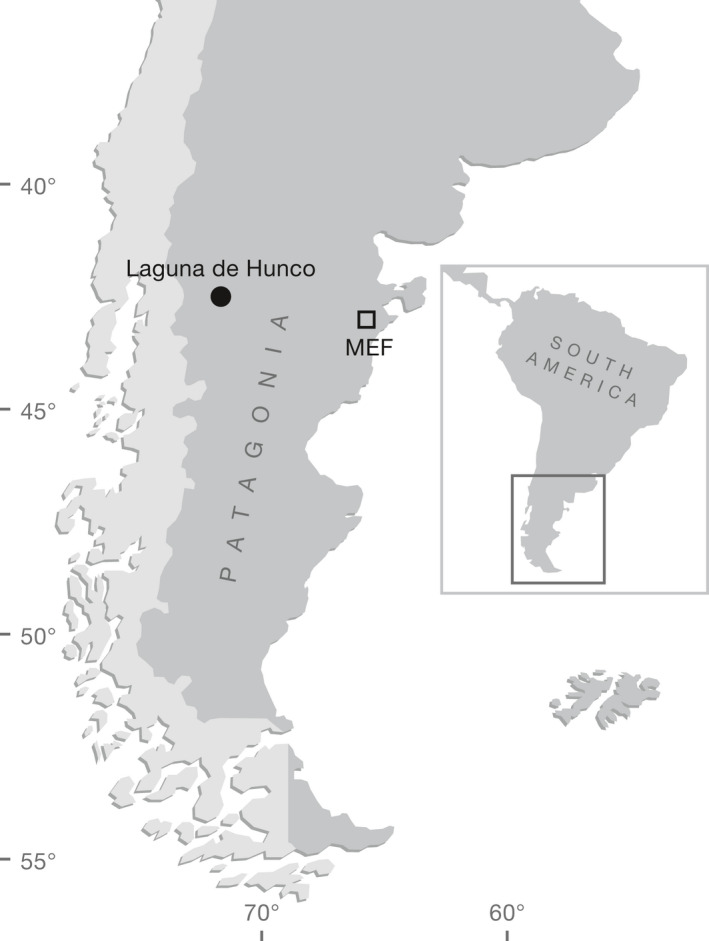
Location of the Laguna del Hunco (LH2) locality, Chubut Province, Patagonia, Argentina. MEF = Museo Paleontológico Egidio Feruglio, Trelew City, Chubut Province, the repository of all fossil specimens reported here.

The Patagonian *Eucalyptus* fossils constitute the oldest record for the genus based on macrofossils and the first confirmed record outside Australia, and demonstrate that the genus had a much broader distribution in the past. Most importantly they predate the estimated age based on molecular data for the origination of crown‐group *Eucalyptus* (Ladiges et al., 2003; Crisp et al., 2004; Thornhill et al., 2015, 2019) by more than 20 million years.

Hermsen et al. (2012) described one of the flowers reported by Gandolfo et al. (2011) as “cf. *Eucalyptus* B” (Fig. 8B in Hermsen et al. 2012; Fig. 2A). This fossil flower has numerous free stamens surrounding the gynoecium in the typical cyclic androecial arrangement of the eucalypts (Fig. 2B). Some of the stamens were preserved completely and consist of filaments and versatile anthers (Fig. 2A). This particular fossil is a compression and some of the anthers include preserved organic material that contained pollen grains. Herein, we report and describe the pollen grains found in situ, evaluate the within‐anther variability of the pollen grains, and assess the phylogenetic position of the fossil when the in situ pollen characters are added to the matrix. In addition, we formally describe the fossil flower based on the new data.

**Figure 2:**
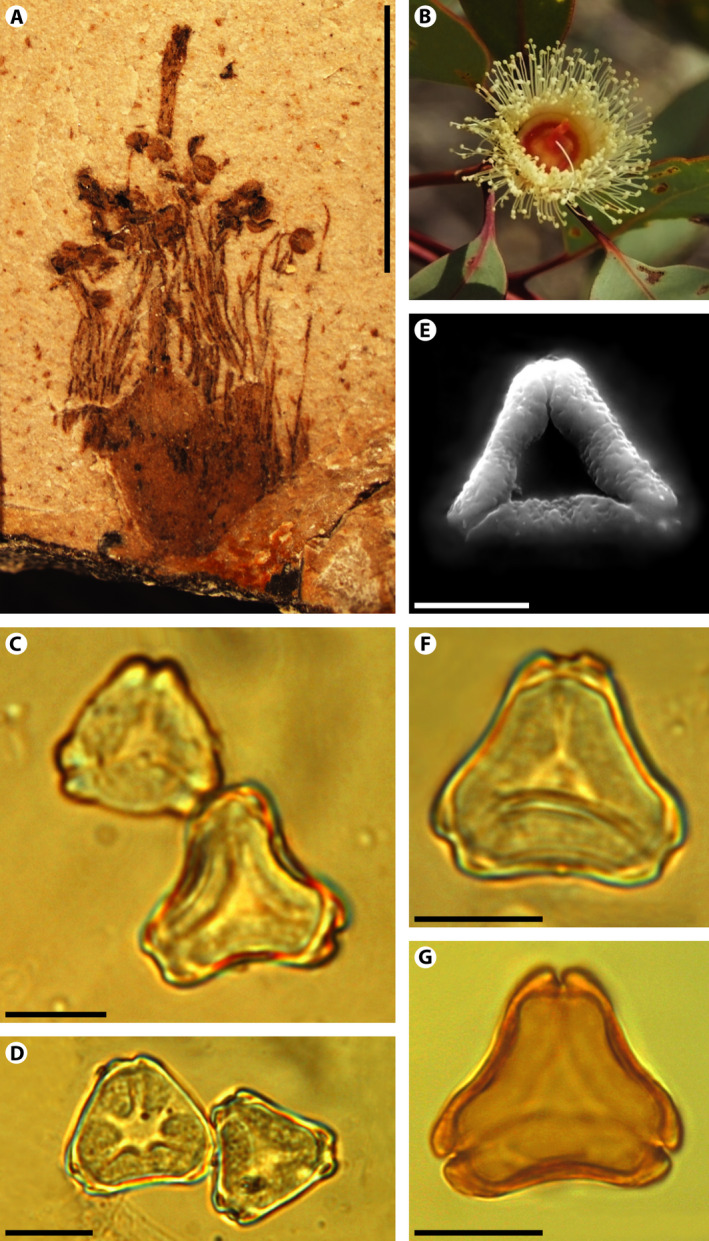
*Eucalyptus xoshemium* Gandolfo and Zamaloa sp. nov., pollen in situ, and extant flower and pollen. (A) Holotype MPEF‐Pb 5022. (B) *Eucalyptus pachyphylla* extant flower (image by Meredith Cosgrove at PlantSystematics.org). (C–F) *Myrtaceidites eucalyptoides* pollen grains found within the anthers (C, D, F with phase contrast, E with SEM). (G) Pollen of extant *Eucalyptus camaldulensis*. Scale bar for (A) = 5 mm, scale bar for (C–G) = 10 µm.

## MATERIALS AND METHODS

### Fossil collection and preparation

The fossil flower was collected at quarry LH2 (Gandolfo et al., 2011; Hermsen et al., 2012) of the Laguna del Hunco locality, Huitrera Formation, that outcrops in Chubut Province, Patagonia, Argentina (Fig. 1). The Laguna del Hunco flora was deposited in sandstones and tuffaceous mudstones representing a lacustrine‐caldera unit of the Chubut River volcanic‐pyroclastic complex (Aragón and Romero, 1984; Aragón and Mazzoni, 1997). The age of the LH2 quarry was calculated as Ypresian, early Eocene (52.22 ± 0.22 Ma) by sanidine dating (Wilf et al., 2003, 2005; Wilf, 2012), which gives a minimum age estimate for the fossils.

The rock matrix of the fossil flower was minimally trimmed in the field and prepared by staff at the Museo Paleontológico Egidio Feruglio (MEF, Trelew, Chubut Province, Argentina) where it was kept at the collection within a box and covered with a plastic bag. The fossil was washed with distilled water for cleaning and to avoid modern pollen contamination. After that, an adhesive cellulose tape was applied over the fossil flower anthers, and once the tape was pulled off, most of the carbonized surface material was transferred to the tape. After that, the surface of the fossil was soaked in acetone and covered with a small cellulose acetate sheet, which was pulled off when it dried up. Both tape and acetate sheet were directly mounted on microscope slides for observation. The slides were studied and photographed using a Nikon Eclipse 80 light microscope with an attached Nikon DS‐L4 camera (Nikon Corp., Minato, Tokyo, Japan) at the MEF. Selected pollen grains were recovered by cutting a portion of the tape for further study under a scanning electron microscopy (SEM). The SEM observations were made with a Philips XL30 TMP microscope (Philips, Amsterdam, North Holland, Netherlands) at the Museo Argentino de Ciencias Naturales Bernardino Rivadavia (MACN), Buenos Aires, Argentina.

The palynological terminology follows that used by Thornhill et al. (2012). The fossil flower and palynological slides are housed at the paleobotanical collection of the Museo Paleontológico Egidio Feruglio, Trelew, Chubut Province, Argentina under the numbers MPEF‐Pb 5022 and MPEF‐Pb 5022 (pollen), respectively.

### Phylogenetic Analysis

For evaluating the newly discovered *Myrtaceidites eucalyptoides* pollen grains within the anther of the fossil *Eucalyptus* flower, we added pollen characters to the single terminal “Patagonian Fossil” of the Gandolfo et al. (2011) combined morphological and molecular matrix (Appendix 3: Combined morphology and molecular data set https://doi.org/10.1371/journal.pone.0021084.s006). The original matrix comprises 18 taxa, including the genus *Lophostemon* (the outgroup) and all the genera of tribe Eucalypteae as follows: *Stockwellia*, *Eucalyptosis*, *Allosyncarpia*, *Arillastrum*, *Corymbia* (with representatives of subgenus *Corymbia*, and of sections Navicularis and Abbreviatae), three species of *Angophora*, six species of *Eucalyptus* (*E. pleurocarpa* and *E. erythrocorys* representing subgenus *Eudesmia; E. camaadulensis* and *E. deglupta* within subgenus *Symphyomyrtus, E. cloeziana* representing subgenus *Idiogenes*, and a single terminal representing *Eucalyptus* subgenus *Eucalyptus*) and the Patagonian fossil species *Eucalyptus xoshemium*.

Additional pollen characters were coded based on Thornhill et al. (2012) without modification except for characters “width” (distance between two corners) and “length”(distance between a corner and the opposite side). Thornhill et al. (2012) considered for each of these two characters seven states as follows: State 0 = 6–10 µm, State 1 = 10–14 µm, State 2 = 14–18 µm; State 3 = 18–22 µm; State 4 = 22–28 µm; State 5 = 28–38 µm, State 6 = 38–50 µm. We modified these by combining them into three states as follows: State 0 = small (6–14 µm), State 1 = medium (15–28 µm), and State 2 = large (29–50 µm) for both characters. Pollen characters and character states newly coded for the Patagonian fossil are: pollen shape (oblate), pore and colpi number (tricolporate), colpal morphology (parasyncolpate with angular colpi), pollen sides (straight, concave or slightly convex), exine pattern (psilate/scabrate), colpal edges (smooth), apocolpal field (psilate), apocolpial island (absent), angles of amb (notched, occasionally rounded), width (medium), and length (medium). Outgroup and modern species were coded directly from Thornhill et al. (2012) except for the “width” and “length” characters as mentioned above.

The resulting morphological matrix was concatenated with the molecular matrix used in Gandolfo et al. (2011) and reanalyzed using parsimony in the program TNT (Goloboff et al., 2008). Multiple advanced search techniques were employed, including the parsimony ratchet (Nixon, 1999), and drift and sectorial searches (Goloboff, 1999), with a range of search parameters (e.g., changing the character sampling scheme for ratchet searches, varied from 5–15%). Each analysis included 200 ratchet iterations, 50 drift iterations, and five rounds of sectorial searches. These runs were repeated more than 20 times, always with the same end results, providing a very high confidence that the resulting trees are the most parsimonious for this data set.

## RESULTS AND DISCUSSION

### Systematics

#### Family

Myrtaceae Jussieu 1789

#### Subfamily

Myrtoideae Sweet 1827

#### Tribe

Eucalypteae Peter G. Wilson 2005

#### Genus

*Eucalyptus* L’Héritier 1789

#### Species

*Eucalyptus xoshemium* Gandolfo and Zamaloa, sp. nov.

Cf. *Eucalyptus* sp. B, Hermsen et al. (2012), Fig. 8B

#### Specific diagnosis

Minute, epigynous, bisexual flower, lacking sepals and petals after anthesis. Hypanthium obconic to campanulate. Androecium of numerous stamens borne in a single cycle surrounding the gynoecium; each stamen with filament and a versatile anther with *Myrtaceidites eucalyptoides* pollen in situ. Ovary inferior, partially embedded within the hypanthium; style solitary; stigma bilobed.

#### Holotype here designated

MPEF‐Pb 5022 (Fig. 2A)

#### Locality and age

Laguna del Hunco, Huitrera Formation, Ypresian (early Eocene), Chubut Province, Argentina, quarry LH2.

#### Specimens examined

MPEF‐Pb 5022 and MPEF‐Pb 5022 (pollen).

#### Etymology

The specific epithet comes from the Tehuelche word “xoshem” that means wind, and refers to the Patagonian strong winds.

#### Measurements

Hypanthium 3 mm high × 4 mm wide, filaments 2–4 mm long, anthers 1 mm long, style 6 mm high.

#### Comments

**Figure 3:**
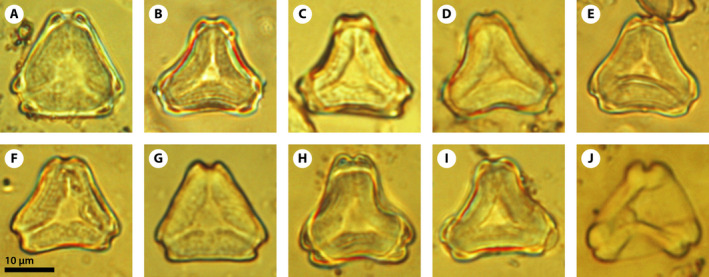
A–J, *Myrtaceidites eucalyptoides* found within the anthers of *Eucalyptus xoshemium* sp. nov. showing the morphological variability in a single individual (MPEF‐Pb 5022 (pollen) with interference contrast).

The *Myrtaceidites* pollen grains found within the anthers of *Eucalyptus xoshemium* sp. nov. are oblate tricolporate angulaperturate and shed as monads, have triangular polar shape, and relatively small size (length: 14.9–22.3 µm; width: 15.8–21.4 µm; Figs. 2C–F, Fig. 3A–J). Their surface ornamentation is faintly granulate or scabrate and the exine is thickened around the endopore enclosing a more or less developed vestibule (Figs. 2C–D, F, 3A–J). The colpi meet near the pole delimitating a triangular apocolpial field (parasyncolpate) with straight sides (angular colpi) and there is no apocolpial island. The pollen sides are straight, concave or slightly convex in polar view (Figs. 2C–F, 3A–J). The apertural apex is mostly notched, occasionally rounded in some specimens.

The pollen grains recovered from anthers of the Patagonian fossil flower are identical to *Myrtaceidites eucalyptoides* Cookson and Pike 1954; in other words, if these pollen grains were found dispersed or isolated in the sediments, they would be confidently identified as *M. eucalyptoides*.

Most Myrtaceae pollen grains are oblate, angulaperturate, and tricolporate, with triangular amb, and small‐to‐medium sized in comparison to other angiosperm families (Thornhill et al., 2012). Pre‐Quaternary pollen that is comparable to modern Myrtaceae is assigned to the fossil genus *Myrtaceidites*, which was first described by Cookson and Pike (1954) and recently reviewed by Thornhill and Macphail (2012). According to that review, the pollen genus encompasses 15 species. Thornhill and Macphail (2012, p. 18) confirmed that *Eucalyptus* sensu stricto is the only living genus known to produce the *M. eucalyptoides* pollen morphotype.

**Figure 4:**
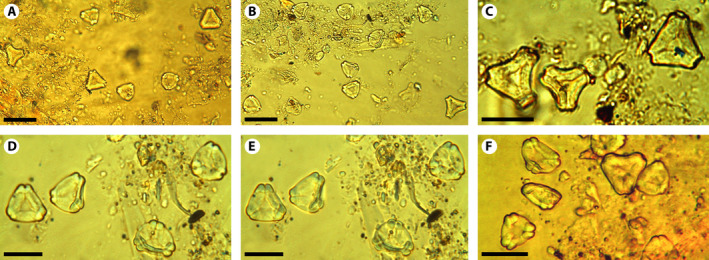
A–F, *Myrtaceidites eucalyptoides*clusters representing the content of the anthers of *Eucalyptus xoshemium*(MPEF‐Pb 5022 pollen; with interference contrast). Scale bar for (A–B) = 40 µm, scale bar for (C–F) = 20 μm. D and E, same pollen grains at different focus.

The discovery of numerous pollen grains (Fig. 4A–F) within the anthers of a single fossil flower allows evaluation of their intraspecific variability by indicating which characters are the most constant and which are not. In this particular case, we see consistency in the small size, the parasyncolpate character of the apertures, the absence of a polar island, the thickening of the exine around the endopores, and the almost psilate‐to‐scabrate exine, whereas the amb shape (notched or rounded), the development of both the vestibule, and the thickening of the wall around the apertures are variable characters. Knowing this intraspecific variability is crucial not only for the characterization of *Myrtaceidites* pollen (especially when found isolated) but also for the interpretation of the fossil pollen record of Myrtaceae in general.

*Myrtaceidites eucalyptoides* is characterized by parasyncolporate grains with strongly thickened nexine around the endopores, and almost psilate exine, variable amb shape, and small‐to‐medium size. Cookson and Pike (1954) recognized two forms of *M. eucalyptoides*: forma *convexus* for all the grains with convex sides and forma *orthus*, which includes those grains with straight or only slightly concave sides in polar view. These authors also suggested that the two forms may represent different groups of species. Nevertheless, both forms are present among the more than 60 pollen grains recovered from the anthers of a single flower, which demonstrates that both forms were produced by the same individual (Figs. 2C–F, 3A–J, 4A–F). Therefore, we can assert that *M. eucalyptoides* forma *convexus* and *M. eucalyptoides* forma *orthus* belong to the same species and that the shape of the sides of the pollen grains in polar view is a variable character of the species. Moreover, all the grains found within the anthers have other features that are highly variable as well, such as the development of the thickening around the endopore, the vestibule formed between the ectocolpus and the endopore, and the angle of the apertures in polar view, which can vary from rounded to truncate (Fig. 3A–J). This remarkable variability could have led to the identification of at least two different species if the grains had been found isolated or dispersed in the sedimentary rocks.

When Hermsen et al. (2012) described the fossil flower and discussed its taxonomical affinities, they suggested the possibility that it could be placed in either *Corymbia* or *Eucalyptus* because it lacks sepals and petals after anthesis and the androecium has several stamens around an ovary, which culminates in a single style. However, the finding of *Myrtaceidites eucalyptoides* pollen grains within its anthers strongly places the fossil flower to crown group *Eucalyptus* and not to stem *Corymbia* or *Angophora. Corymbia* pollen grains are much larger in size, have apocolpial islands, and are syndemicolpate or parasyncolpate (Thornhill et al., 2012); and, actually the fossil species *Myrtaceidites tenuis* (Harris 1965) is the pollen type linked to the genera *Corymbia* and *Angophora* (Thornhill and Macphail, 2012). The *Myrtaceidites* grains found in *E. xoshemium* are small, have endopores with a thickened exine, and an almost psilate exine, which are features of most *Eucalyptus* pollen (Thornhill et al., 2012). They are even remarkably similar to pollen produced by modern species of *Eucalyptus* (Fig. 2G).

It is worth mentioning that *Eucalyptus xoshemium* sp. nov. and *E*. *frenguelliana* Gandolfo and Zamaloa leaves, which are indistinguishable from those of modern *Eucalyptus* (Gandolfo et al., 2011; Hermsen et al., 2012) were found at the same locality (LH2).

### Phylogenetic analysis

**Figure 5:**
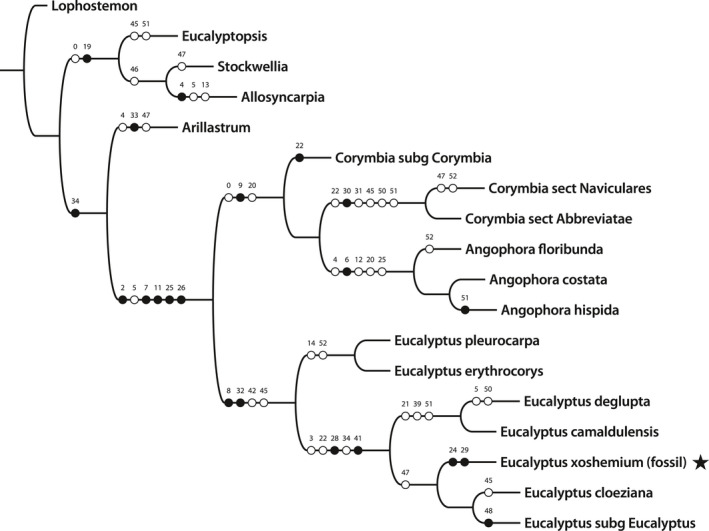
Strict consensus tree of two equally most parsimonious trees, showing the position (black star) of *Eucalyptus xoshemium*, the Patagonian fossil, nested within *Eucalyptus* sensu stricto. Only morphology and pollen characters are mapped (black dots represent characters that are not homoplasious changes; white dots represent characters that are homoplasious changes). Length: 409 steps, CI: 0.64, RI: 0.76.

Parsimony analysis of the combined matrix with floral morphology, molecular sequence data, and added pollen characters resulted in two most parsimonious trees. The phylogenetic position of the fossil, when the in situ pollen characters are added, remains the same as the position of the original analysis of the fossil flower without pollen (Gandolfo et al., 2011). We obtained two equally most parsimonious trees. The consensus tree is 409 steps long, a consistency index (CI) of 0.64, and a retention index (RI) of 0.79; it is presented in Figure 5. The placement of the fossil with new data validates the original conclusion that the fossil flower and the enclosed pollen belong in the extant genus *Eucalyptus*.

### Fossil record

The *Eucalyptus* fossil record has been extensively treated by several authors throughout the past few decades, and it was recently discussed and summarized in the light of new evidence by Hermsen et al. (2012) and Hill et al. (2016), and references therein. From these reviews, it is clear that macrofossils from Australia and New Zealand (mostly wood, leaves, and reproductive structures) cannot be placed unequivocally in one genus among the three that make up Eucalypteae because of relative scarcity, poor preservation, or lack of adequate revision. Therefore, the majority of those macrofossils has been placed only within *Eucalyptus* sensu lato (Hill et al., 2016) and are referred to as “eucalypt” fossils. The oldest reliable and accepted *Eucalyptus* sensu stricto macrofossils are the ones from the early Eocene Laguna del Hunco, Patagonia (Gandolfo et al., 2011; Hermsen et al., 2012), and *Eucalyptus xoshemium* (the flower herein described) is among them. This record demonstrates the presence of *Eucalyptus* well outside of its current distribution area in Australasia (with one additional recently arrived species in Mindanao, Philippines; Ladiges et al., 2003).

Until now, *Myrtaceidites eucalyptoides* has been only sporadically found in sediments of middle Eocene and younger age in southern Australia, in one Neogene locality in Burundi, Africa (pending revision), in the broadly dated Oligocene–Lower Pliocene Forest Bed of the Falkland Islands (presumed contaminant by Macphail and Thornhill, 2016; see Thornhill and Macphail, 2012 for citations on the localities) and in the Neogene of New Zealand (Raine et al., 2011). The finding of *M. eucalyptoides* within the anthers of *Eucalyptus xoshemium* sp. nov. represents the oldest and, so far, the only confirmed record for South America, expanding its previously known stratigraphical distribution and definitely setting its age to at least the early Eocene. Furthermore, the discovery of the flower in association with the exquisitely preserved leaves, as previously mentioned, strongly suggest that they were deposited close to the source and with minimum or no transport, reinforcing their autochthonous origin. Therefore, the pollen grains found within the flower’s anthers have an intrinsic stratigraphic value because the flower is restricted to a level (LH2 quarry) with excellent chronological control in the stratigraphic sequence (see Materials and Methods).

### Age of the tribe Eucalypteae and of *Eucalyptus*

Dating molecular phylogenies has become essential to comprehend the evolution of taxa, but to produce robust calibrated phylogenies, it is necessary to have reliable calibration points. Even though there are several eucalypt calibrated phylogenies, the estimated age of the tribe Eucalypteae (the eucalypts) is still controversial (see Bayly, 2016; Hill et al., 2016, and citations therein). Crisp et al. (2005) argued that for eucalypts calibration can be “problematic because no well‐dated fossil that can be placed accurately on a tree is known.” Ladiges et al. (2003) and Crisp et al. (2004) suggested that the eucalypts originated at least in the Late Cretaceous (~70 Ma) whereas Thornhill et al. (2015, 2019) and Macphail and Thornhill (2016) disagreed with those authors and proposed the divergence time of the tribe was early‐to‐early late Early Eocene (~55.2–51.2 Ma). The confirmed phylogenetic position of *Eucalyptus xoshemium* (and therefore of *Myrtaceidites eucalyptoides* as well) in *Eucalyptus* sensu stricto strongly suggests that the genus *Eucalyptus* had begun to diversify before the early Eocene. Therefore, the split of the eucalypts from the rest of the Myrtaceae must have been before the early Eocene, as suggested by Ladiges et al. (2003) and Crisp et al. (2004). Taking this into account, the age of this fossil also implies that the date for the origin of the stem linage of *Eucalyptus* must predate its estimated age based on molecular data.

## CONCLUSIONS

As previously stated, biases in the fossil record have been abundantly addressed in the literature (e.g., Gandolfo et al., 2008; Xing et al., 2015, and citations therein); nevertheless, it is important to remark that although it is uncommon to find fossils in organic connection, sometimes these occasional discoveries are exceptionally informative because they represent unique and direct evidence that different organs belong to the same organism and that, at a certain level, they allow a total or partial reconstruction of the organism that produced them. Hence, the inclusion of such fossils within a phylogenetic context gives stronger robustness to the phylogenetic conclusions. Until the current time, there was no direct evidence that *Myrtaceidites* pollen was produced by *Eucalyptus*, and the presence of *M. eucalyptoides* outside Australia was seriously challenged (Macphail and Thornhill, 2016). Indeed, all current assumptions about which genera these pollen morphotypes belong to come from comparisons of fossil pollen with their modern analogs. Macphail and Thornhill (2016) stressed the fact that the recognition of eucalypt pollen in the fossil record can be difficult because the pronounced thickening of the nexine around the endopores (the primary morphological character used to identify fossil eucalypts) “is observable in *Myrtaceidites eucalyptoides* (modern affinity *Eucalyptus*) but does not occur in the pollen morphospecies considered to represent the earliest eucalypt‐type in Australia, namely *M. tenuis* (modern affinity *Angophora, Corymbia*)” (Macphail and Thornhill, 2016, p. 587). Furthermore, these fossils provide critical information on within‐anther variability of *M. eucalyptoides* pollen grains that is relevant to understanding intraspecific variation, because they were obtained from a single individual.

As a whole, the suite of *Eucalyptus* fossils found in Patagonia fills the gap between the micro‐ and macrofossil record of this iconic genus. The finding of *Myrtaceidites eucalyptoides* within the anthers of a fossil *Eucalyptus* flower is the first recorded organic connection between organs for the genus and for the family Myrtaceae. And, the fact that independently both the flower and the pollen have been linked to *Eucalyptus* strongly suggests that the morphological characters of the flower and of the pollen were evolving at the same time and in parallel. In addition, because the age of *Eucalyptus xoshemium* is well‐dated to the Ypresian (early Eocene, ~52.2 Ma), it is precisely the “type of fossil” necessary for any phylogenetic study on the eucalypts.

In summary, this is the oldest known record of *Myrtaceidites eucalyptoides* worldwide, it proves that its earliest recorded appearance predates previously suggested ages for *Eucalyptus*, and that the *Eucalyptus* lineage was present in Patagonia at least by the early Eocene. The fossil pollen found in situ within the anthers of the *E. xoshemium* is consistent with the genus *Eucalyptus*, and adding the pollen characters to the combined molecular and morphological matrix do not change the most important result—the inclusion of the fossil in the crown group of modern *Eucalyptus*. These new findings provide crucial information for understanding patterns and tempo of the evolution of the eucalypts in general, and in particular of *Eucalyptus* s.s. Undoubtedly, these fossils are a perfect example on the importance of paleobotanical work when addressing evolutionary processes in deep time.

## AUTHOR CONTRIBUTIONS

M.C.Z., M.A.G., and K.C.N. conceived the study, described, and analyzed fossil data; M.C.Z. extracted and studied the pollen grains; K.C.N. and M.A.G. analyzed phylogenetic data. M.A.G. led the writing of the manuscript with major contributions from M.C.Z. and K.C.N.

## Data Availability

Combined morphology and molecular data set can be accessed at https://doi.org/10.1371/journal.pone.0021084.s006‐ Gandolfo et al (2011). The fossil specimen and palynology slides are curated in perpetuity at Museo Paleontológico Egidio Feruglio, Trelew City, Argentina.
